# Gamification in Apps and Technologies for Improving Mental Health and Well-Being: Systematic Review

**DOI:** 10.2196/13717

**Published:** 2019-06-26

**Authors:** Vanessa Wan Sze Cheng, Tracey Davenport, Daniel Johnson, Kellie Vella, Ian B Hickie

**Affiliations:** 1 Brain and Mind Centre The University of Sydney Sydney Australia; 2 Queensland University of Technology Brisbane Australia

**Keywords:** well-being, video games, gamification, mental health, health behavior, systematic review, eHealth, mHealth, health informatics

## Abstract

**Background:**

There is little research on the application of gamification to mental health and well-being. Furthermore, usage of gamification-related terminology is inconsistent. Current applications of gamification for health and well-being have also been critiqued for adopting a behaviorist approach that relies on positive reinforcement and extrinsic motivators.

**Objective:**

This study aimed to analyze current applications of gamification for mental health and well-being by answering 3 research questions (RQs). RQ1: which gamification elements are most commonly applied to apps and technologies for improving mental health and well-being? RQ2: which mental health and well-being domains are most commonly targeted by these gamified apps and technologies? RQ3: what reasons do researchers give for applying gamification to these apps and technologies? A systematic review of the literature was conducted to answer these questions.

**Methods:**

We searched ACM Digital Library, CINAHL, Cochrane Library, EMBASE, IEEE Explore, JMIR, MEDLINE, PsycINFO, PubMed, ScienceDirect, Scopus, and Web of Science for qualifying papers published between the years 2013 and 2018. To answer RQ1 and RQ2, papers were coded for gamification elements and mental health and well-being domains according to existing taxonomies in the game studies and medical literature. During the coding process, it was necessary to adapt our coding frame and revise these taxonomies. Thematic analysis was conducted to answer RQ3.

**Results:**

The search and screening process identified 70 qualifying papers that collectively reported on 50 apps and technologies. The most commonly observed gamification elements were *levels or progress feedback*, *points or scoring*, *rewards or prizes*, *narrative or theme*, *personalization*, and *customization*; the least commonly observed elements were *artificial assistance*, *unlockable content*, *social cooperation*, *exploratory or open-world approach*, *artificial challenge*, and *randomness*. The most commonly observed mental health and well-being domains were anxiety disorders and well-being, whereas the least commonly observed domains were conduct disorder and bipolar disorders. Researchers’ justification for applying gamification to improving mental health and well-being was coded in 59% (41/70) of the papers and was broadly divided into 2 themes: (1) promoting engagement and (2) enhancing an intervention’s intended effects.

**Conclusions:**

Our findings suggest that the current application of gamification to apps and technologies for improving mental health and well-being does not align with the trend of positive reinforcement critiqued in the greater health and well-being literature. We also observed overlap between the most commonly used gamification techniques and existing behavior change frameworks. Results also suggest that the application of gamification is not driven by health behavior change theory, and that many researchers may treat gamification as a *black box* without consideration for its underlying mechanisms. We call for the inclusion of more comprehensive and explicit descriptions of how gamification is applied and the standardization of applied games terminology within and across fields.

## Introduction

### Conceptualizing Gamification

Gamification is the application of gameful elements for nongame purposes. Although the term has, on occasion, been used interchangeably [1] with the closely related concept of *serious games* (video games developed for a primary purpose other than player enjoyment [2]), both concepts are examples of *applied games*, which involve the implementation of “design concepts and qualities from the game world” [3]. Despite being a relatively new example of applied games, gamification has received considerable interest from the health research community for its potential to increase engagement with health interventions and motivate behavior change [4-8]. However, it should not be assumed that any intervention automatically incorporating gamification will have increased engagement [4]. Even the commonly cited ability of gamification to provide fun and engaging experiences cannot be taken for granted, as fun does not necessarily translate to increased motivation to engage [9]. Nonetheless, proponents of gamification point to its potential cost-effectiveness, accessibility, and flexibility, as well as the increasing worldwide popularity of video games and the potential of gamification to increase intrinsic motivation [6,10,11], as reasons to apply it to health and well-being.

Multiple definitions have been proposed for the term *gamification*, including the “use of game design elements in non-game contexts” by Deterding et al [12], and “a process of enhancing a service with affordances for gameful experiences in order to support user’s overall value creation” by Huotari and Hamari [13]. They each provide a guiding framework through which to conceptualize it, with different definitions fitting different usage and research contexts. For example, Huotari and Hamari’s definition of gamification emphasizes how it can be used to enhance existing services, such as mental health and well-being interventions, and the mechanisms through which they work. It is a useful way to conceptualize gamification when *implementing* it for mental health and well-being purposes and is arguably more compatible with the general goals of health research.

On the other hand, the definition by Deterding et al is more useful for operationalizing gamification. By emphasizing the contrast between playfulness (*paidia*) and gamefulness (*ludus*), Deterding et al categorize gamification as games-based in part form, comparable but distinct to serious games (games-based in whole form) and playful design (play-based in part form), and the conceptual opposite to toys (play-based in whole form). This definition also prioritizes game design elements, implying a taxonomical approach useful for piecing out the individual elements of gamification and operationalizing the various ways it can be applied. This makes this definition useful for *studying* gamification.

### Gamification for Health and Well-Being

Recent reviews find that gamification is most commonly applied to physical fitness interventions and to motivate health behaviors for managing chronic illnesses, and although gamified mental health and well-being interventions exist, they are less common [6,7]. This may be due to the inappropriateness of applying common gameful elements (points, rewards, achievements, social comparison, and competition) to mental health, especially in circumstances where users could potentially be in distress [14,15]. According to self-determination theory, humans are intrinsically motivated to satisfy their basic psychological needs of autonomy, competence, and relatedness [16]. As subjective enjoyment of video games has been empirically linked to the satisfaction of these constructs [17], gamification should, in theory, also be compatible with increasing intrinsic motivation. However, many instances of gamification for general health and well-being rely on positive reinforcement and extrinsic motivators [6], an approach that has been criticized [18,19]. There may be an understandable reluctance in the community to extend what is perceived to be a behaviorist implementation of gamification [20] to mental health and well-being domains.

Admittedly, by definition, it is more straightforward to influence intervention users’ extrinsic motivation than their intrinsic motivation. However, organismic integration theory (OIT) posits that there are low-autonomy and high-autonomy variants of extrinsic motivators [16], with low-autonomy variants having the most harmful effect on intrinsic motivation [9]. The ideal implementation of gamification would, therefore, harness intrinsic motivation and the types of extrinsic motivation that are most likely to be internalized, such as identified or integrated regulation [16]. Previous research in the health field has expanded on properties of video games that may be more compatible with intrinsic motivation, such as narrative, fantasy, and interactivity [21-23]. These properties may also be associated with improved emotional intelligence and regulation [24].

Recent reviews also report a lack of explicit linkage between the theory and application of gamification [1,25]. Although gamification elements have been theoretically matched to behavior change techniques [4,26], this has not translated to theory-driven gamification [22]. Furthermore, even when behavior change theory is referenced, its implementation may not be as comprehensive as it could be [22]. Although most calls for gamification and its application for health and well-being (including mental health and well-being) invoke motivational reasons [4-7,27,28], *motivation* is only 1 driver of health behavior change. According to the behavior change wheel by Michie et al [29], the other 2 are *capability* and *opportunity*, and it is these drivers that gamification may be missing its potential to support [22].

### Operationalizing Gamification

It is difficult to review past gamification research when the word *gamification* means slightly different things across papers. For example, in the review of health and fitness mobile phone apps by Lister et al [22], *gamification* is used to cover the concepts of leaderboards, levels, digital rewards, tangible prizes, competitions, and social pressure but not avatars or “narrative context” (as Lister et al consider them “game elements”). However, Johnson et al use *gamification* to describe all these elements in their review [6]. Similarly, although Brown et al incorporate both narrative and avatars in their gamification element *story/theme* [30], both Johnson et al and Lister et al separate these features into 2 elements. In another example, Sardi et al combine *feedback/rewards* into 1 game mechanic [7], whereas other reviews consider these features separately [6,22,25,30]. In addition, few reviews define their gamification elements. This makes comparison of findings across reviews difficult without in-depth examination of individual review methodology.

The term *game* is notoriously difficult to define [31], which may contribute to why definitions of *gamification* vary considerably. Although games have always been present in culture in varying forms [32], the current bidirectional trend of games influencing culture and vice versa (the *ludification of culture* and the *cultivation of ludus* [33]; gamification being a clear example of the latter) may explain the increasing interest shown by researchers in technologies and components that are not inherently game-like but are culturally associated with video games, such as virtual reality, augmented reality, and avatars [1]. Although the fuzzy boundaries of the term *gamification* point to the enthusiasm people from varying fields have for adopting it and may encourage creativity in games (and gamification) research [31], they also represent a clear challenge for the study of gamification.

In their recent review, Seaborn and Fels recommend that the intentional use of gamification be a key indicator of whether an app or technology can be defined as containing gamification or not [1]. This accounts for the fact that certain elements of gamification, such as social comparison and progress feedback, are also present in other behavior change frameworks such as persuasive systems design [34] and prevents false positives from being identified. It also corresponds with Huotari and Hamari’s definition of gamification as enhancing a basic service provided by the app or technology [13] (in this case, improving mental health and well-being).

### Study Aims

Previous systematic reviews on gamification in health and well-being (including mental health and well-being) have narrowed foci (eg, on evaluation [6] and adherence [30]), resulting in a somewhat incomplete picture of the implementation of gamification for mental health and well-being across the fields of medicine, psychology, computer science, and other related fields. In addition, it has been 4 years since the most recent comprehensive database search [7] was conducted. This study, therefore, aims to conduct an updated systematic review of the application of gamification in apps and technologies for improving mental health and well-being, with a focus on breadth, and using a more in-depth taxonomy of gamification elements. To ensure maximum relevance given rapidly changing technology, only studies from the past 5 years were considered. This review aimed to answer the following research questions (RQs):

- RQ1: Which gamification elements are most commonly applied to apps and technologies for improving mental health and well-being?
- RQ2: Which mental health and well-being domains are most commonly targeted by these gamified apps and technologies?
- RQ3: What reasons do researchers give for applying gamification to apps and technologies for improving mental health and well-being?

## Methods

### Search Strategy and Screening Process

A pilot search was conducted in March 2018 to assess the feasibility of this study. This search was replicated on November 21, 2018, by 1 author (VWSC), who searched the databases ACM Digital Library, CINAHL, Cochrane Library, EMBASE, IEEE Explore, JMIR, MEDLINE, PsycINFO, PubMed, ScienceDirect, Scopus, and Web of Science with the following search string: (gamif* OR gameful* OR “game-based”) AND (“mental health” OR “wellbeing” OR “well-being” OR “mental illness” OR “mental disorder”). This string was adjusted to match each database’s requirements. All citations were screened according to the following 5 steps:

1. Initial search: All citations were downloaded to a citation manager (Endnote X8) library file;
2. Probable inclusion: A preliminary screen was performed according to the inclusion criteria;
3. Prune duplicates: All remaining references were collated into 1 group, and duplicates were removed;
4. Definite inclusion: All remaining references were stringently assessed against inclusion criteria;
5. Additional literature: Additional literature was extracted from reference lists of review papers identified in the initial search (step 1), the reference lists of citations from step 4 (eg, for other papers reporting on the same app or technology), and the pilot search. Those that satisfied the inclusion criteria were added to the final dataset.

### Inclusion Criteria

Identified citations had to satisfy the following main inclusion criteria to qualify for inclusion:

1. Be published between the years 2013 and 2018.
2. Describe an app or technology related to the improvement of mental health and well-being outcomes (including secondary outcomes).
3. Define their app or technology as being *gamification*, *gamefulness*, or *game-based.*
4. Be in the English language.
5. Not be labeled a serious game.

The search string was kept general to create a wide search net; however, there are limitations to this approach, which are discussed later in this paper. Apps or technologies that were labeled as *serious games* were not included, as serious games are complete games and, therefore, fall outside the scope of this review. Some citations identified in the initial search appeared to use the terms *gamification* and *serious game* interchangeably, pointing to the inconsistent use of terminology observed by previous reviews of gamification [1]. These citations were individually discussed by 2 authors (VWSC and KV), with reference to Huotari and Hamari’s definition of gamification (“a process of enhancing a service with affordances for gameful experiences in order to support user’s overall value creation”) [13], until agreement was reached on whether to include or exclude them from the final dataset. Specifically, we considered apps or technologies that appeared to be complete games specifically developed for their purpose as serious games and excluded them from the dataset as a result.

The search also identified many primarily physical health interventions. They were included if assessing an aspect of mental health and well-being as a research outcome (whether primary or secondary).

### Coding Process

Due to its breadth, we used the taxonomy of gameful elements by Tondello et al [35] as a foundation for answering RQ1. Similarly, we used the mental and substance use disorders categories from the Global Burden of Disease study [36] as a starting point for answering RQ2. For RQ3, we coded reasons whenever they appeared in the body of the papers in our dataset. The dataset was coded with QSR International’s NVivo 11, a computer-assisted qualitative data analysis software.

Using the above-identified frameworks as a starting base, 2 authors (VWSC and KV) read and re-read the first 10% of papers to assess the preliminary coding frame and identify any further emergent codes. Both authors also independently coded the first 10% of papers based on the preliminary coding frame. Discrepancies between coders were discussed and resolved, and the coding frame was updated with more precise definitions for each gamification element. This process was repeated on the next 10% of papers until sufficient interrater agreement (κ=0.758) was reached.

The coding process was not straightforward because of an overlap in terminology. This was particularly the case for RQ1. For example, although the term *levels* is commonly used to refer to advancing progress, as in *leveling up*, it is also used to refer to new, more difficult environments [1]. Similarly, we observed *points* being used as both progress markers (experience points) and currency. This necessitated significant revisions between the preliminary and final coding frames. Furthermore, although certain categories of gameful elements identified by Tondello et al [35] were well represented in our dataset (eg, *customization*), others were almost nonexistent (eg, *altruism*). To maintain a balance between simplicity and detail, we collapsed less used gameful element categories into 1 code (eg, collapsing all of Tondello et al’s *assistance* elements into our gamification element *artificial assistance*) while keeping other, more used gameful elements separate (eg, maintaining the distinction between *social competition*, *social networking*, *social cooperation*, and *social comparison*). Similar gameful elements that were commonly observed together (such as *levels and progression* and *progress feedback*; or *narrative or story* and *theme*) were also grouped together and precisely defined in our coding frame. Our final coding frame containing 18 gamification elements and 17 mental health and well-being domains is presented in Multimedia Appendix 1.

One author (VWSC) then applied this coding frame to the entire dataset, including returning to earlier coded papers and recoding when necessary. Data extracts from each new paper were compared with the previously coded data extracts for all relevant codes, and against the coding frame, to ensure consistency.

### Thematic Analysis of Research Question 3 Codes

One author (VWSC) collated and thematically analyzed [37] all data extracts pertaining to RQ3. As mentioned previously, coded data extracts were compared against each other multiple times during coding rounds, and across multiple rounds, to refine the coding frame, ensure consistency, and further delineate the concepts covered by each code.

As RQ3 investigates researchers’ motivations, coding was done using an interpretivist approach, with the aim of staying relatively close to the dataset, that is, the article text was prioritized over the coder’s higher-level interpretations. This was done to prevent the coder from imposing additional meaning that the authors of the original papers may not have intended [38]. For example, although the codes *Increase engagement with intervention* and *Increase motivation to use* may appear to cover similar concepts (although not identical [9]), they were coded separately, as we observed multiple instances of those particular wordings. All RQ3 codes and example quotes are also presented in the final coding frame in Multimedia Appendix 1.

## Results

### Search Strategy and Screening Process

**Figure 1:**
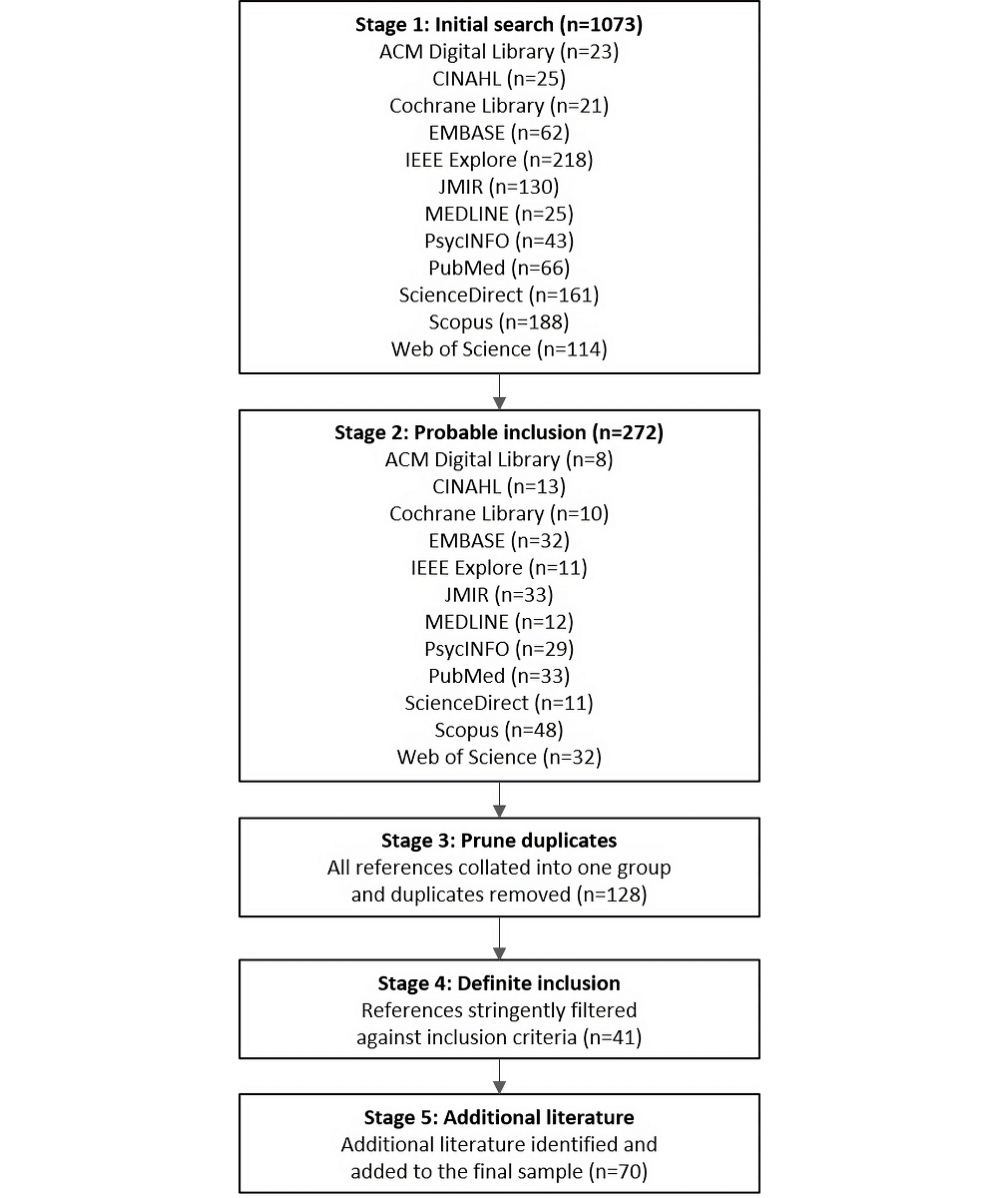
Flowchart of articles identified by the search and screening process.

Figure 1 shows a summary of the search and screening process.

At the end of the screening process, 70 qualifying papers were identified, collectively reporting on 50 apps and technologies. Multimedia Appendix 2 presents a summary of each app or technology, including description, mental health and well-being domain(s), and gamification elements.

### Research Question 1: Gamification Elements

**Figure 2:**
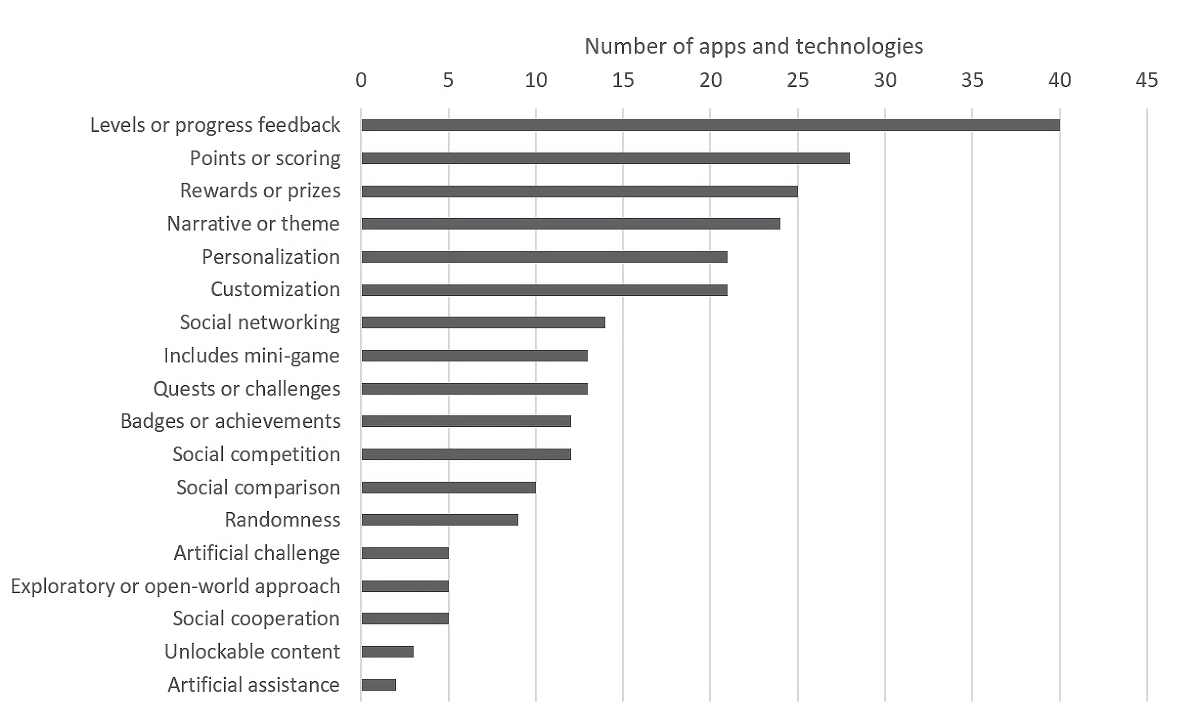
Number of apps and technologies containing the specified gamification elements.

Figure 2 shows the number of apps and technologies in the dataset that contain the specified gamification elements.

Of the 18 gamification elements, the most commonly coded were *levels or progress feedback* (40/50, 80%), *points or scoring* (28/50, 56%), *rewards or prizes* (25/50, 50%), *narrative or theme* (24/50, 48%), *personalization* (21/50, 44%), and *customization* (21/50, 44%). The least commonly coded elements were *artificial assistance* (2/50, 4%), *unlockable content* (3/50, 6%), *social cooperation* (5/50, 10%), *exploratory or open-world approach* (5/50, 10%), *artificial challenge* (5/50, 10%), and *randomness* (9/50, 18%).

**Figure 3:**
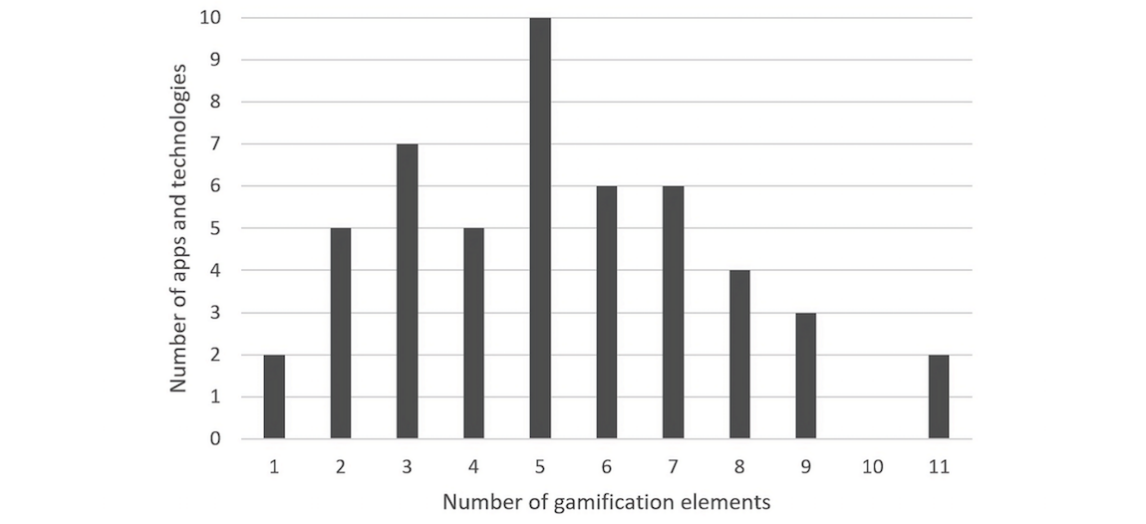
Number of gamification elements coded in each app or technology.

Figure 3 shows the distribution of the number of gamification elements coded in 1 app or technology, with the mode and median value being 5.

### Research Question 2: Mental Health and Well-Being Domains Targeted by Gamification

**Figure 4:**
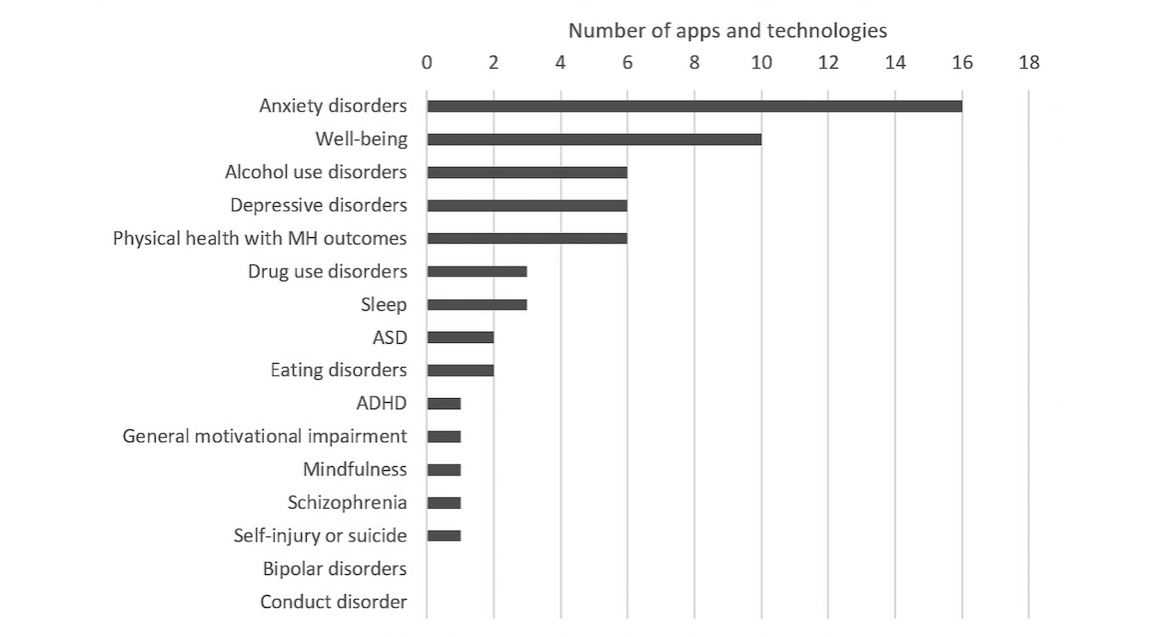
Number of apps and technologies targeting the specified mental health and well-being domains. ASD: autism spectrum disorders; ADHD: attention-deficit/hyperactivity disorder; MH: mental health.

Figure 4 shows the count of mental health and well-being domains that were represented in the dataset. (The count does not sum to 50, as some apps or technologies targeted multiple mental health and well-being domains.)

Of the 17 mental health and well-being domains, the most commonly coded were anxiety disorders (16/50; 32%), well-being (10/50, 20%), alcohol use disorders (6/50, 12%), depressive disorders (6/50, 12%), and physical health with mental health and well-being outcomes (5/50, 12%). The least commonly coded were conduct disorder (0/50, 0%), bipolar disorders (0/50, 0%), self-injury or suicide (1/50, 2%), schizophrenia (1/50, 2%), mindfulness (1/50, 2%), general motivational impairment, (1/50, 2%), and attention-deficit/hyperactivity disorder (1/50, 2%).

### Research Question 3: Reasons for Applying Gamification to Improving Mental Health and Well-Being

**Figure 5:**
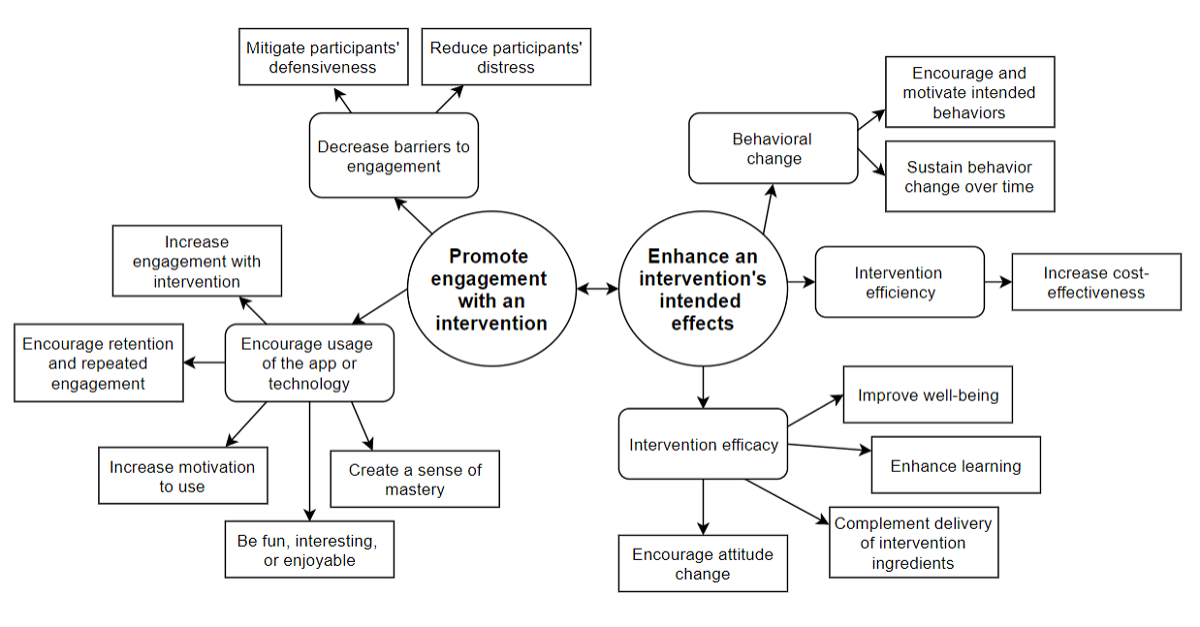
Thematic diagram showing themes, subthemes, and codes.

We found justification for applying gamification to improving mental health and well-being in 41 of the 70 papers (59%) in the dataset. Figure 5 shows the organization of themes, subthemes, and codes, with themes in ovals, subthemes in rounded rectangles, and codes in rectangles. Example quotes for each code are presented in the coding frame in Multimedia Appendix 1.

The codes were sorted into 5 main subthemes, which were further sorted into 2 main themes: (1) *promoting engagement with an intervention* and (2) *enhancing an intervention’s intended effects*.

Of the 5 subthemes, 2 fell under the first theme (promoting engagement): (1) *encouraging usage of their app or technology* and (2) *decreasing barriers to engagement*. The former was the most commonly cited reason for using gamification and was coded in 31 of the 41 papers (76%), whereas the latter was much less prevalent (6/41 papers, 15%).

The remaining 3 subthemes fell under the second theme (enhance an intervention’s intended effects): (1) *behavior change*, (2) *intervention efficiency*, and (3) *intervention efficacy*. Of these subthemes, *behavior change* was the most commonly coded (14/41 papers, 32%), followed by *intervention efficacy* (12/41 papers, 29%) and *intervention efficiency* (2/41 papers, 5%).

## Discussion

### Summary

The search and screening process identified 70 qualifying papers that collectively reported on 50 apps and technologies. Of the 18 gamification elements in our coding frame, the most commonly coded gamification elements were *levels or progress feedback*, *points or scoring*, *rewards or prizes*, *narrative or theme*, *personalization*, and *customization*, whereas the least commonly coded were *artificial assistance*, *unlockable content*, *social cooperation*, *exploratory or open-world approach*, *artificial challenge*, and *randomness*. The mode count of gamification elements coded in the included apps and technologies was 5.

Of the 17 mental health and well-being domains in our coding frame, the most commonly coded were anxiety disorders and well-being, whereas the least commonly coded were conduct disorder, bipolar disorders, self-injury or suicide, schizophrenia, mindfulness, general motivational impairment, and attention-deficit/hyperactivity disorder.

Finally, researchers’ justification for applying gamification to apps and technologies for improving mental health and well-being was coded in 59% (41/70) papers. In these 41 papers, we identified 2 main themes: (1) *promoting engagement with an intervention* and (2) *enhancing an intervention’s intended effects*.

### Research Question 1: Gamification Elements

We observed *levels or progress feedback* in a vast majority (40/50, 80%) of the apps and technologies that aim to support the improvement of mental health and well-being in our dataset, making it the most commonly applied gamification element. This is consistent with previous reviews of both the academic literature [7] and stress management apps in the Google Play Store [25] and is unsurprising, as in addition to being easy to implement, progress feedback is a key behavior change technique [4,26]. The near-ubiquity of this element may also point at the influence personal informatics has had on health technology [6].

Many critics of gamification point to the inadequacy of the *points, badges, and leaderboards* approach [39] in targeting intrinsic motivation and creating satisfying user experiences [6,18]. But although these elements were present in the apps and technologies in our dataset, only *points or scoring* was in the top 5. This contradicts earlier findings that points are used rarely for mental health and well-being [30] and may be due to the difference in inclusion criteria between both reviews. Alternatively, it could be due to developments in the field of health gamification, with recent mental health and well-being apps and technologies drawing on learnings from early adopter health fields such as physical activity and chronic illness. Meanwhile, *badges or achievements* were observed in 12/50 (24%) apps and technologies. We did not code leaderboards as a specific gamification element, instead including it into our broader gamification element *social competition*, which we observed in 12/50 (24%) apps and technologies. These results suggest that in mental health and well-being domains, points, badges, and leaderboards are far from dominant and that alternative models of gamification are being applied.

Previous research has outlined the potential of progress feedback, points, and rewards to promote behavior change [40,41]; however, their effectiveness is unclear [42] and may depend on how these elements are designed to fit the basic intrinsic and extrinsic motivational processes underlying the app or technology [6,9]. On the other hand, evidence for the potential of *personalization* and *customization* (conceptually similar to the term *tailoring* commonly used in health behavior change literature) is more promising [8,43]. Tailoring offers users increased levels of autonomy, which, according to OIT, would contribute to increased likelihood of internalized motivation and well-being [16]. Ultimately, however, more research is required to establish whether these improvements persist in the long term or merely result from novelty effects [4].

Of the 50 apps and technologies in our dataset, the mode count of gamification elements was 5 (10 apps and technologies, 20%), with the distribution shaped similar to a bell curve with mild positive skew (Figure 3). Our observed mode is much greater than the mode of 1 element identified in previous reviews of Web-based mental health interventions whose evaluation also assessed adherence [30] and stress management apps in the Google Play Store [25]. This finding aligns with recent research showing that a greater diversity of types of rewards in a game led to greater presence, enjoyment, and effort [44]. The fact that our study’s coding frame contains more gamification elements than the study by Brown et al [30] may contribute to this difference; however, as Hoffmann et al’s study [25] coded for 17 gamification techniques, this may not be the only reason. Furthermore, the increased range (1-11) of gamification elements observed in our sample of apps and technologies for improving mental health and well-being suggests that researchers may be growing more comfortable with applying a range of gamification elements for mental health and well-being [30]. Previous calls for the inclusion of more gamification elements in health and well-being interventions [22] may have also contributed to this increase.

Most of the more frequently observed gamification elements in our dataset, namely progress feedback, points, rewards, personalization, badges, quests, and varying social features, overlap with those in other behavior change frameworks [4,26]. Notably, in persuasive systems design, these features are named self-monitoring, praise, rewards, tailoring, recognition, goal setting, normative influence, cooperation, competition, and social comparison [34]. These overlapping elements make up the bulk of our observations in our dataset, with the exception of *social cooperation*, which we observed infrequently in our dataset (5/50, 10%), *includes mini-game* (13/50, 26%), and *narrative or theme*, which is not found in other behavior change frameworks but was one of the most commonly observed gamification elements in our dataset (24/50, 48%). In its current state, the application of gamification to improving mental health and well-being seems difficult to distinguish from approaches stemming from other behavior change frameworks such as persuasive systems design.

So what distinguishes gamification from these approaches? What added value does gamification offer compared with other behavior change frameworks and techniques? The answer may lie in the gamification elements we observed less frequently in our dataset: *randomness*, *artificial challenge*, *exploratory or open-world approach*, *social cooperation*, *unlockable content*, and *artificial assistance*. Although elements such as *artificial challenge* and *artificial assistance* are likely underutilized because of their usefulness only in certain contexts (eg, dynamic difficulty adjustment to create a state of flow during attentional bias modification training (ABMT) [45] or providing facial identification cues in an attention training intervention for children on the autism spectrum [46]), other elements such as *randomness*, *exploratory or open-world approach*, and *social cooperation* may be more complementary to mental health and well-being in general.

*Randomness*, which is one of the key types of play and games according to Caillois [32], can be implemented via a random reinforcement schedule, for example, to facilitate learning [47]. However, more integral to this gamification element is the anticipation of not knowing exactly what to expect, for example, by offering intervention participants missions that have been randomly drawn from a larger pool of missions [48] and the sense of excitement that comes with it. Similarly, designing mental health and well-being interventions to accommodate an *exploratory or open-world approach* complements the flexibility of contemporary internet experiences and may even be expected by intervention participants [27]. However, it may be challenging to apply this to therapeutic approaches whose structures may be more rigid, such as cognitive behavioral therapy. A possible solution in cases similar to these could be to make all modules immediately accessible but indicate a recommended module order [14]. In this way, the user’s autonomy is not thwarted [16], and they are empowered with the knowledge of how to navigate the intervention in a way that can benefit them most.

Despite the clearly demonstrated benefits of social connectedness on well-being [49], *social cooperation* is underutilized in mental health and well-being interventions, particularly in comparison with other social elements such as *comparison* and *competition*. Social cooperation represents a positive way of interacting with others that does not explicitly place value on all involved parties (as it would through competition or comparison) and is a way to satisfy our innate need for relatedness and promote well-being [16]. Despite this, the only instances of social cooperation we observed in our dataset were in physical activity and well-being interventions, with the majority in the form of cooperation nested within competition (cooperating with team members to compete against other teams). As this approach is still competitive at its core, it may be incompatible with many mental health and well-being domains [14,15]. Most instances of social support we observed in our dataset were instead in the form of *social networking*, where users of an app or technology could interact with and affirm each other through posts, private messages, and gifts. It may be useful to draw inspiration from cooperative mechanics from commercial video games to identify how best to apply social cooperation to improving mental health and well-being in more task- and domain-compatible ways. For example, in Massively Multiplayer Online games or video games such as *Snipperclips* [50], players can work together to achieve a system-defined goal (effectively players vs system). Applied to an ABMT intervention, a system-defined social cooperation goal could be having all members of a team complete a stage a certain number of times or collectively achieve a certain score. Designers of mental health and well-being interventions can also consider integrating real-time location data into their functionality (eg, an app aimed at decreasing levels of social anxiety challenges its users to call a gym and provides the phone number of a nearby gym [51]). A recent example of a successful app with this functionality is Pokémon GO [52], which encourages its users to make meaningful connections with physical locations and people [53].

### Research Question 2: Mental Health and Well-Being Domains Targeted by Gamification

Anxiety disorders was the most commonly targeted mental health and well-being domain in our dataset (16 apps and technologies, 32%), followed by well-being (10 apps and technologies, 20%). Of note is the fact that no gamified apps and technologies targeting bipolar disorders and conduct disorder were identified in this review. (We did, however, exclude 1 intervention aimed at preventing substance abuse and relationship violence for being a serious game [54].) As these domains, particularly bipolar disorders, have significant associated global burden of disease [36], this may be a research gap worth targeting.

Overall, there is a greater level of diversity in mental health and well-being domains compared with that in previous reviews of the literature [6,7,30]. However, more work remains to be done not only in designing engaging and efficacious gamified mental health and well-being interventions but also in evaluating their effectiveness. The slow pace of clinical research is directly at odds with the fast pace of technological change, frequently rendering interventions obsolete in the time taken to establish their efficacy. For this reason, nontraditional development and evaluation methods such as agile development and rapid prototyping may be more suitable for gamified mental health and well-being interventions [27]. However, care must be taken to ensure that no harm, particularly from the application of gamification [1,55], is caused to intervention testers during these stages of development and testing.

### Research Question 3: Reasons for Applying Gamification to Improving Mental Health and Well-Being

#### Theme 1: Promoting Engagement With an Intervention

*Encouraging usage* of the app or technology was the dominant reason for applying gamification, appearing in 31 of the 41 (76%) papers that provided a reason for using gamification. Gamification was purported to improve multiple aspects of engagement, including fun and enjoyableness, and create a sense of mastery. This would encourage both first contact and repeated contact with the app or technology, concepts that are analogous to engagement and retention. However, further research is needed to learn how gamification enhances engagement [4] and whether it may be more effective at establishing initial engagement or ongoing use.

Gamification was also said to be a tool to *decrease barriers to engaging* with an intervention, both in terms of mitigating participants’ defensiveness and reducing participants’ distress. This was much less used, appearing in 6 (15%) papers. The mental health and well-being domains represented were also limited, with mitigating participants’ defensiveness mentioned only by authors of interventions targeting alcohol use and anxiety disorders [56-59] and reducing participants’ distress exclusively mentioned by authors of interventions targeting phobia [60,61]. Interestingly, despite significant societal levels of stigma against mental health problems, these reasons were not cited for any other mental health and well-being domain.

#### Theme 2: Enhancing an Intervention’s Intended Effects

Most of the data extracts under this theme related to *behavior change* (14 papers, 32%). Specifically, researchers aimed to use gamification to encourage intended behaviors and sustain behavior change over time. This was unsurprising, given the focus of these apps and technologies on improving mental health and well-being through behavior change, possibly in response to academic calls for action [4,5]. The ability of gamification to support behavior change is also somewhat supported by existing research [41,62,63], although further research is required on whether, and how for long, these effects persist [4].

Other aims related to *intervention efficacy* were also mentioned, including encouraging attitude change, enhancing learning, improving well-being, and using gamification elements to complement the delivery of intervention ingredients (eg, by presenting an ABMT task as a game of snap [64]). As mentioned previously, more research is needed to establish the extent of the effects gamification may have on supporting these goals.

Finally, although this was only mentioned in 2 (5%) papers, gamification was touted as a way to potentially increase the cost-effectiveness of interventions either by attracting users to participate without using material incentives or by making the feedback and reward loop interesting enough so that the intervention attracted new users and incentivized the existing users to continually generate new content, creating a closed loop [58]. This specific intervention design was for a personalized normative feedback intervention targeting problematic levels of alcohol consumption and may, therefore, be impractical for many mental health and well-being domains requiring trained moderators and therapists. However, designers of more self-directed initiatives such as preventive or well-being interventions (particularly those that rely on social comparison) may find this a useful model.

#### Overall

Of 41 papers, 13 (32%) explicitly linked gamification to motivation to use the app or technology. This points to the origins of gamification as being defined as a motivational affordance and the way it was initially introduced to electronic health and mobile health as a tool to increase engagement and motivate behavior change [4-6]. However, in the context of behavior change theory, motivation is only 1 driver of behavior change, with the other 2 being capability and opportunity [29]. The focus on motivation, seemingly at the expense of capability and opportunity [22], represents lost potential.

Some gamification elements are particularly compatible with capability and opportunity. For example, according to Lister et al, capability can be promoted via *self-monitoring*, and opportunity by providing *cues to action* and *peer pressure* [22]. The gamification equivalent to these techniques would be *levels or progress feedback*, *personalization*, and social mechanics (whether *competition*, *cooperation*, *networking*, or *comparison*) respectively. As an example, a mental health and well-being app or technology might identify certain times of day when users are engaged in particular activities or have free time and time notifications accordingly (*personalization*).

Finally, although the above sections provide insights into what the writers of some papers in our dataset intended to achieve with gamification, it is important to note that reasoning behind the decision to implement gamification was only provided in 41 of 70 (59%) of papers. This may be indicative of a lack of consideration of the mechanisms through which gamification may influence behavior change in a large portion of mental health and well-being–related research. Furthermore, this may indicate that the lack of linkage between the theory and application of gamification observed in the greater literature [1,25] is also present, to a degree, in mental health and well-being. In other words, some applications of gamification to apps and technologies for improving mental health and well-being may be treating gamification as a *black box*, which is clearly problematic. With reference to Huotari and Hamari’s definition of gamification [13], designers of mental health and well-being interventions may find it helpful to identify the key attitude and behavior change mechanisms and processes through which they intend the intervention to work and how these interact with established evidence-based techniques in their field. Once these core *services* (or *intervention principles* [65]) are identified, gamification can then be applied in various ways to enhance these services. This would result in a more targeted, theory-driven, evidence-based application of gamification to improving mental health and well-being.

### Study Limitations

This study aimed to systematically review literature published from 2013 to 2018 to identify any and all instances of the application of gamification to apps and technologies for improving mental health and well-being. Furthermore, this review had a broad focus, including sources that are traditionally excluded in systematic reviews such as conference papers and conceptual papers. This was done to ensure as much accuracy as possible in describing the current state of the gamification of health and well-being. However, there are some limitations to this study’s methodology that must be acknowledged to fully contextualize our results.

First, there is a possibility that some qualifying papers were not identified by the search. Although a wide variety of keywords were used to capture as many results as possible, this may particularly be the case for more specialist papers that may only discuss their specific mental illness and not include the phrases *mental health*, *wellbeing*, *well-being*, *mental illness*, or *mental disorder*. Furthermore, interventions were frequently described as *gamification* when they were actually (as judged by the authors) serious games. Although those studies were excluded, the initial search would not have captured any interventions that were the other way around—gamified interventions that were labeled *serious games*. The search process would also have failed to identify apps or technologies that the academic literature does not report and explicitly link to gamification, including many commercially developed apps or technologies. The results of this review are, therefore, not fully generalizable to commercially developed apps and technologies for improving mental health and well-being.

It is also likely that not all gamification elements in an intervention were able to be coded, as researchers may not have fully described their apps and technologies in the papers. It is also possible that the gamification elements described in papers may have been removed from the app or technology in subsequent software updates. Furthermore, in some studies, as in best practice, gameful design was so embedded within the intervention that the gamification elements could not be separated from the active ingredients of the intervention. Alternatively, and on the other extreme, in some cases, the only mention of gamification was a blanket statement that it had been applied, making it difficult to judge which elements these statements referred to. For these reasons, *all* features of an intervention were evaluated against the coding frame and coded. In doing so, coding detail was also kept consistent between studies that only evaluated 1 version of an intervention and studies that evaluated a control version against a gamified version (of which there were not many). As the health gamification field matures, there is a need for the inclusion of more detail when describing the implementation of gamification and more consistent use of applied games terminology within and across fields.

As certain intervention paradigms can be used for multiple contexts (eg, ABMT), the range of mental health and well-being domains we observed is also likely to be an underestimate of the range of domains, both within mental health and well-being and across all aspects of health and well-being, to which gamification can potentially be applied. Similarly, as the focus of this review was the *improvement* of mental health and well-being, apps and technologies that used gamification for other mental health and well-being–related purposes such as measurement were excluded from the review.

Finally, as the primary aim of this review is to provide a record of all reported gamified apps and technologies for improving mental health and well-being within the past 5 years (2013-2018), we did not collect any information on the included apps or technologies’ efficacy or effectiveness on any evaluation metric. However, given that a previous review has identified the relative lack, and low level of quality, of evidence for the effectiveness of gamification for health and well-being [6], this analysis may be premature. Furthermore, although this review investigates individual gamification elements, we acknowledge and argue that gamification is best implemented and evaluated holistically, as implied by Huotari and Hamari’s definition of gamification [13]. Instead of evaluating individual gamification elements, a more practical and informative approach may be to evaluate individual (gamified) intervention principles [65].

### Conclusion and Future Directions

This paper reports the results of a systematic review on all applications of gamification to improving mental health and well-being reported in the literature in the past 5 years (2013-2018). A total of 12 databases and journals were searched for qualifying papers, and from the search results, we identified 50 qualifying apps and technologies. Results suggest that gamification is being applied to a greater range of mental health and well-being domains compared with previous reviews and that a greater diversity of gamification elements is being used. Our results also suggest that in the context of improving mental health and well-being, gamification is not being implemented in the behaviorist fashion focusing mostly on positive reinforcement that has been observed and criticized in the wider literature [19]. Importantly, however, our results are only reflective of gamified apps and technologies reported within the academic literature. Future research can conduct a review of commercially developed gamified apps and technologies for improving mental health and well-being and compare and contrast findings with those derived from the academic literature. Similar reviews can also be conducted for serious games and commercial games (potentially including both video games and nondigital games) with mental health themes.

This review also found that certain gamification elements, such as *randomness*, *artificial challenge*, *artificial assistance*, *exploratory or open-world approaches*, and *social cooperation*, are underutilized for the improvement of mental health and well-being and that further research (ideally with rapid prototyping methods such as agile development [27]) is needed to identify how best these elements can be applied to improving mental health and well-being, if at all. There is also a need to consider and evaluate how gamification may promote a wider variety of drivers for health behavior change. Although current applications of gamification in improving mental health and well-being are primarily for improving motivation (to engage with the intervention or change behavior), future applications should consider how gamification can serve other behavior change drivers such as capability and opportunity [22,29]. It is also important to evaluate whether gamification may lead to unintentional, harmful effects, and in what circumstances this may occur [1,55]. For example, what would be the effects and ethical implications of using randomness in a substance use disorder intervention, given that they both involve dopamine?

Finally, most researchers in health technology probably share the fundamental goal of developing interventions that enable and empower the greatest improvement in health and well-being for the greatest number of people in the target population. To achieve this, there is an urgent need to describe the implementation of gamification to health interventions in more explicit and precise detail and to standardize applied games terminology (including *gamification*, *serious games*, and other types of applied games) within and across fields. It may also be fruitful to take a step back from the single-minded focus on engagement that has been characteristic in academic literature on the gamification of health and well-being until now [6,30], and consider more broadly how gamification can enhance the basic functionality of a mental health and well-being intervention. Identifying a mental health and well-being intervention’s goals and intentionally designing gamification to support them in novel and pragmatic ways may be the best way to achieve rapid progress in this field.

## Appendix

Multimedia Appendix 1 Final coding frame for gamification elements (Research Question [RQ] 1), mental health and well-being domains (RQ2), and reasons for applying gamification for improving mental health and well-being (RQ3).

Multimedia Appendix 2 Summary of each app or technology, including description, mental health and well-being domain(s), and gamification elements.

## Abbreviations

ABMT: attentional bias modification training
OIT: organismic integration theory
RQ: research question
