# Supplementary material for: Gamification in Apps and Technologies for Improving Mental Health and Well-Being: Systematic Review
Source: JMIR Ment Health. 2019 Jun 26;6(6):e13717. doi: 10.2196/13717 (PMC6617915; doi:10.2196/13717)
Supplement: Multimedia Appendix 1 [file mental_v6i6e13717_app1.pdf]

Coding frame: RQ1 (gamification elements)

**Gameful element**	**Definition**
Artificial assistance	Assistance imposed or facilitated by the system, within the system experience. Doesn't include things like allowing repeats, but includes things like decreasing difficulty of completing tasks.
Artificial challenge	Have difficulty imposed by the system (for example, via limited lives, increased speed, or time pressure).
Badges or achievements	Being awarded permanent visual recognition, for example via badges, certificates and achievements, by the system in response to accomplishing meaningful goals inside the system.
Customization	Being able to customize avatars and other types of representation (of the self or of the player character), or other aspects of the user experience, either freely or through spending in-game currency or real currency.
Exploratory or open-world approach	User is free to explore the system at their leisure and access to system components or modules is not restricted.
Includes mini-game	A smaller game is included in the app or technology but it remains a sub-component (i.e. app or technology is not a serious game).
Levels or progress feedback	User is given an indication of their progress in a task and in the overall content of the system, and how far they have to go to succeed in or finish the task and reach the next milestone.
Narrative or theme	The system adopts a certain theme or premise and presents itself faithfully according to that premise (e.g. a CBT program may be presented on a game board; results may be presented on a spinning wheel), and may or may not include an underlying storyline.
Personalization	The system learns about you either by asking you directly or by analyzing your behavior in the system, and adapts what and how it presents to you to suit you (e.g. adaptive difficulty, using real-time location data). As opposed to 'Customization', this is driven by the system.
Points or scoring	The user gains points for completing tasks, which can be either static or scale based on their performance.
Quests or challenges	Users are invited to complete specific tasks in exchange for rewards.
Randomness	Having the result of a certain aspect of the system (quests, tasks, etc.) be determined by chance, either partially or entirely. Includes scripted events (i.e. the appearance of chance).
Rewards or prizes	Receiving rewards or prizes by completing specific tasks or goals, or by reaching certain milestones.
Social comparison	The system allows users to see other users' actions, and to show off their own achievements, progress and/or status to other users.
Social competition	Users are placed in implied (via leaderboard) or direct competition with each other.
Social cooperation	Working together with other users, for example as part of a team, to achieve a goal.
Social networking	System or app allows users to make connections with each other, perhaps facilitating this by matching similar users, and to express or act upon these connections, for example via gifting, or communicating via comments, messages, stickers, etc.
Unlockable content	Unlocking additional content (outside of the main game or intervention) after reaching certain milestones, achieving a significant goal, or exploring different paths, etc.

Cheng VWS, Davenport T, Johnson D, Vella K, Hickie IB. (2019). Gamification in Apps and Technologies for Improving Mental Health and Well-Being: A Systematic Review. JMIR Mental Health

Coding frame: RQ2 (mental health and well-being domains)

Alcohol use disorders
Anxiety disorders
Attention-deficit/hyperactivity disorders
Autistic spectrum disorders
Bipolar disorders
Conduct disorder
Depressive disorders
Drug use disorders
Eating disorders
General motivational impairment
Mindfulness
Physical health with mental health and well-being outcomes
Schizophrenia
Self-injury or suicide
Sleep
Well-being

Coding frame: RQ3 (reasons for applying gamification to improving mental health and well-being)

Theme	Subtheme	Code	Example quote
Promote engagement with an intervention	Decrease barriers to engagement	Mitigate participants' defensiveness	"It was anticipated that providing feedback in the context of a game, which was not explicitly focused on alcohol, might improve efficacy among heavier drinkers by reducing defensive reactions to the intervention content." [1]
		Reduce participants' distress	"In the context of [virtual reality exposure therapy], which can be highly distressing for patients, gamification may reduce the negative experiences of treatment." [2]
	Encourage usage	Be fun, interesting, or enjoyable	"The rehabilitation games were designed to combine a variety of rehabilitation exercises with gaming elements, thus making the otherwise monotonous practice more competitive, motivating, interesting and enjoyable." [3]
		Create a sense of mastery	"Digital coins and badges give the users a sense of accomplishment […]" [4]
		Encourage retention and repeated engagement	"The design of this mindfulness project exercises several common game mechanics to maintain user interest over time, without attempting to distract them during mindfulness meditation." [5]
		Increase engagement with intervention	"The Wellbeing Game uses gamification to encourage engaging in the Five Ways to Wellbeing through." [6]
		Increase motivation to use	"Similar to the ARET system, the VRET system will also follow a gamification approach to motivate the patient during her/his therapy by feeding back the actual achievements but also the progress that is already accomplished." [7]
Enhance an intervention's intended effects	Behavior change	Encourage and motivate intended behaviors	"In this paper we explore how a gamified digital behaviour change intervention can be adapted to encourage people of different personality types to engage in simple acts of kindness." [8]
		Sustain behavior change over time	"Gamification also can be useful for clients with initiation impairments due to frontal lobe deficits by acting as an adaptive aid to prompt behavior and develop habits and routines." [9]
	Intervention efficacy	Complement delivery of intervention ingredients	"Because the ultimate goal of PNF interventions is to induce behavior change by helping participants learn facts about the true prevalence of various behaviors among their peers, CampusGANDR was designed to reward participants for accurate perceptions of peers' behaviors using a system of points." [10]
		Encourage attitude change	"[…] the present study explored the effects of gamification on […] creating positive attitudes towards sleeping and waking up at optimal times." [11]
		Enhance learning	"To enhance learning and motivation, the program calibrated the difficulty level between each round, depending on the participant's performance." [12]

		Improve well-being	"Through gamification, many benefits, such as the commitment of users to participate in various activities and sharing of experiences that have potential well-being effects on the users may be reached." [13]
	Intervention efficiency	Increase cost-effectiveness	"Delivering a brief alcohol intervention within the context of a gamified app allows the intervention to be disguised as something fun and interesting, which may reduce or even eliminate the need to provide compensation in order to attract and retain participants." [10]

1. Earle AM, LaBrie JW, Boyle SC, Smith D. In pursuit of a self-sustaining college alcohol intervention: Deploying gamified PNF in the real world. Addictive Behaviors. 2018 2018/05/01/;80:71-81. doi: https://doi.org/10.1016/j.addbeh.2018.01.005.
2. Lindner P, Miloff A, Hamilton W, Reuterskiöld L, Andersson G, Powers MB, et al. Creating state of the art, next-generation Virtual Reality exposure therapies for anxiety disorders using consumer hardware platforms: design considerations and future directions. Cognitive Behaviour Therapy. 2017 2017/09/03;46(5):404-20. doi: 10.1080/16506073.2017.1280843.
3. Shin J-H, Ryu H, Jang SH. A task-specific interactive game-based virtual reality rehabilitation system for patients with stroke: a usability test and two clinical experiments. Journal of NeuroEngineering and Rehabilitation. 2014 2014/03/06;11(1):32. doi: 10.1186/1743-0003-11-32.
4. Pramana G, Parmanto B, Lomas J, Lindhiem O, Kendall CP, Silk J. Using Mobile Health Gamification to Facilitate Cognitive Behavioral Therapy Skills Practice in Child Anxiety Treatment: Open Clinical Trial. JMIR Serious Games. 2018 05/10;6(2):e9. doi: 10.2196/games.8902.
5. Choo A, May A, editors. Virtual mindfulness meditation: Virtual reality and electroencephalography for health gamification. 2014 IEEE Games Media Entertainment; 2014 22-24 Oct. 2014.
6. Keeman A, Näswall K, Malinen S, Kuntz J. Employee Wellbeing: Evaluating a Wellbeing Intervention in Two Settings. Frontiers in Psychology. 2017 2017-April-04;8(505). doi: 10.3389/fpsyg.2017.00505.
7. Ben-Moussa M, Rubo M, Debracque C, Lange W-G. DJINNI: A Novel Technology Supported Exposure Therapy Paradigm for SAD Combining Virtual Reality and Augmented Reality. Frontiers in psychiatry. 2017;8:26-. PMID: 28503155. doi: 10.3389/fpsyt.2017.00026.
8. Ciocarlan A, Masthoff J, Oren N, editors. Kindness is contagious: Study into exploring engagement and adapting persuasive games for wellbeing. 2018: Association for Computing Machinery, Inc.
9. Fox LG, Barnes D. BoosterBuddy: Using Gamification as a Compensatory Strategy for Motivational Deficits. Psychiatric Services. 2016;67(1):141-2. doi: 10.1176/appi.ps.661101.
10. Boyle SC, Earle AM, LaBrie JW, Smith DJ. PNF 2.0? Initial evidence that gamification can increase the efficacy of brief, web-based personalized normative feedback alcohol interventions. Addictive Behaviors. 2017 2017/04/01/;67:8-17. doi: https://doi.org/10.1016/j.addbeh.2016.11.024.
11. Ilhan E, Sener B, Hacihabiboğlu H. Creating awareness of sleep-wake hours by gamification. In: Tscheligi M, De Ruyter B, Meschtscherjakov A, Fuchsberger V, Murer M, Tscheligi M, editors.: Springer Verlag; 2016. p. 122-33.
12. Pieters EK, De Raedt R, Enock PM, De Putter LMS, Braham H, McNally RJ, et al. Examining a Novel Gamified Approach to Attentional Retraining: Effects of Single and Multiple Session Training. Cognitive Therapy and Research. 2017 2017/02/01;41(1):89-105. doi: 10.1007/s10608-016-9803-z.
13. Ihamäki P, Heljakka K, editors. The sigrid-secrets geocaching trail: Influencing well-being through a gamified art experience. 2017: CEUR-WS.

Cheng VWS, Davenport T, Johnson D, Vella K, Hickie IB. (2019). Gamification in Apps and Technologies for Improving Mental Health and Well-Being: A Systematic Review. JMIR Mental Health
