# Supplementary material for: Gamification in Apps and Technologies for Improving Mental Health and Well-Being: Systematic Review
Source: JMIR Ment Health. 2019 Jun 26;6(6):e13717. doi: 10.2196/13717 (PMC6617915; doi:10.2196/13717)
Supplement: Multimedia Appendix 2 [file mental_v6i6e13717_app2.pdf]

Intervention	Description	Mental health domain	Gamification elements
0Phobia [1]	Self-directed virtual reality (VR) exposure therapy mobile app for acrophobia used with cardboard VR goggles. The gamified component involves acting as a theatre employee who is tasked to spot objects from various heights.	Anxiety disorders	Narrative or theme
Active Team [2]	A physical activity intervention delivered through a mobile app aiming to encourage users to undertake physical activity. The social gamified component includes gifting, leaderboards, and challenges.	Physical health with mental health and well-being outcomes	Customization<br>Levels or progress feedback<br>Quests or challenges<br>Social competition<br>Social networking
Agoraphobia Free [3]	Mobile app delivering interactive game-based cognitive behavioral therapy, tailored specifically towards agoraphobia and panic. The app takes the premise of the user guiding a virtual character through therapy sessions and instructs the user to apply the learned techniques to their own situation.	Anxiety disorders	Narrative or theme
Beat the Street [4-7]	A community intervention where individuals and teams compete to tap at as many radio-frequency identification (RFID) scanners as possible. These scanners are stylized as 'Beat Boxes' and are positioned all over a town or city. Participants are assigned to teams and teams compete to score the most points. A few individual participants are also randomly chosen to receive prizes every week.	Physical health with mental health and well-being outcomes	Games of chance<br>Levels or progress feedback<br>Narrative or theme<br>Points or scoring<br>Rewards or prizes<br>Social comparison<br>Social competition<br>Social cooperation<br>Social networking
BoosterBuddy [8]	A mental health tracking mobile app that aims to motivate users to perform self-management and behavioral activation tasks through the premise of completing quests to wake up their 'buddy'. Through using the app, users unlock options to aesthetically customize their buddy.	General motivational impairment	Customization<br>Levels or progress feedback<br>Narrative or theme<br>Personalization<br>Points or scoring<br>Quests or challenges<br>Rewards or prizes<br>Unlockable content

Cheng VWS, Davenport T, Johnson D, Vella K, Hickie IB. (2019). Gamification in Apps and Technologies for Improving Mental Health and Well-Being: A Systematic Review. JMIR Mental Health

BRANCH [9]	An electronic screening and brief intervention mobile app containing a drink diary, goal-setting functions, information and resources on reducing drinking, and normative drinking feedback. The app is enhanced by social features and a point-based system.	Alcohol use disorders	Customization<br>Personalization<br>Points or scoring<br>Social competition<br>Social networking
CampusGANDR [10, 11]	A personalized normative feedback intervention aimed at reducing alcohol use in undergraduate students. It is presented as a social game where users are ostensibly matched with other Facebook users of the same age and gender and can compare their behavior on various aspects of university life.	Alcohol use disorders	Customization<br>Games of chance<br>Levels or progress feedback<br>Narrative or theme<br>Points or scoring<br>Rewards or prizes<br>Social comparison<br>Social competition
Challenger App [12, 13]	A gamified mobile cognitive behavioral therapy app aimed at social anxiety disorder. Users identify skills they want to gain and are assigned challenges to complete, which are personalized to the user. Users can also leave each other anonymous feedback and advice.	Anxiety disorders	Badges or achievements<br>Customization<br>Levels or progress feedback<br>Narrative or theme<br>Personalization<br>Quests or challenges<br>Rewards or prizes<br>Social comparison<br>Social networking
Cheese Ninja Game [14]	A gamified cognitive bias modification training paradigm. The study contained two gamified versions, one with an additional social component and one without. Codes are based on the social gamified version.	Alcohol use disorders	Narrative or theme<br>Personalization<br>Social comparison<br>Social networking
CityBuilder Game [15]	A cognitive bias modification training task (specifically visual probe task; VPT) targeting substance misuse, embedded into a city-building game. Users earn points from completing VPT rounds that they can use between rounds to customize their city and can view and rate the cities of other participants.	Alcohol use disorders<br>Drug use disorders	Customization<br>Levels or progress feedback<br>Points or scoring<br>Social comparison<br>Social networking
Coping with Voices [16, 17]	A computerized cognitive behavioral therapy program aimed at helping users cope with auditory hallucinations.	Schizophrenia	Includes mini-game<br>Social comparison

Crush the Crave [18-20]	An evidence-based mobile app for smoking cessation, aimed at young adult smokers.	Physical health with mental health and well-being outcomes	Badges or achievements<br>Customization<br>Includes mini-game<br>Levels or progress feedback<br>Personalization<br>Social competition<br>Social networking
Daily Challenge [21]	An online intervention delivered via a website and email focusing on improving general well-being (physical, emotional, social, etc.) Participants are set an email every day with a daily challenge to complete a small task related to improving their health and well-being.	Well-being	Badges or achievements<br>Customization<br>Levels or progress feedback<br>Points or scoring<br>Quests or challenges<br>Rewards or prizes<br>Social comparison<br>Social cooperation<br>Social networking
DJINNI [22]	A design concept for an exposure therapy system targeting social anxiety disorder that can be delivered through either augmented reality or virtual reality. Participants are encouraged to progress in their treatment through real-time indicators of their progress.	Anxiety disorders	Artificial assistance<br>Levels or progress feedback<br>Personalization<br>Points or scoring<br>Rewards or prizes
Emotiv EEG headset [23]	Virtual reality software providing users natural, relaxing virtual environments (e.g. Angkor Wat) to facilitate mindfulness meditation.	Mindfulness	Levels or progress feedback<br>Points or scoring<br>Unlockable content
Empowered Brain system [24]	A smart glasses-based intervention that aims to improve attention and social communication deficits in children. One module, Face2Face, rewards staring at faces through cartoon images and arrows, and another, Emotion Charades, contains a 2-player emotion identifying game.	Attention-deficit/ hyperactivity disorders<br>Autistic spectrum disorders	Artificial assistance<br>Levels or progress feedback<br>Personalization<br>Points or scoring<br>Rewards or prizes
IMPACT - 'Intrinsically-Motivating Playable Attentional Control Training' [25]	An attentional bias modification paradigm aimed at reducing attention bias for negative stimuli (in this case disgusted human faces). Users are scored based on performance. The system also adapts the task difficulty based on the user's performance.	Anxiety disorders<br>Depressive disorders	Artificial challenge<br>Levels or progress feedback<br>Personalization<br>Points or scoring

IntelliCare suite [26]	A suite of mobile apps that each represent and encourage a different behavioral strategy or skill (e.g. goal setting, sleep hygiene) useful for treating depression and anxiety.	Anxiety disorders<br>Depressive disorders	Badges or achievements<br>Customization<br>Exploratory or open-world approach<br>Levels or progress feedback<br>Personalization
Kindness is Contagious [27-29]	A persuasive intervention aimed at improving subjective well-being. In this 7-day intervention, participants are assigned 'kindness activities' daily that are personalized based on their personality type.	Well-being	Levels or progress feedback<br>Personalization<br>Points or scoring<br>Quests or challenges<br>Rewards or prizes<br>Social competition<br>Social networking
Learn to Quit [30]	A mobile smoking cessation app that delivers acceptance and commitment therapy to its users. It is tailored for people with serious mental illness and delivers its content via modules containing a simple narrative.	Physical health with mental health and well-being outcomes	Badges or achievements<br>Includes mini-game<br>Levels or progress feedback<br>Narrative or theme<br>Personalization<br>Rewards or prizes
MEBook [31, 32]	A multimedia intervention (a learning environment with gaming elements) that aims to deliver social greetings training via video self-modelling to children with autistic spectrum disorders.	Autistic spectrum disorders	Levels or progress feedback<br>Narrative or theme<br>Personalization
MindMax [33-37]	An Australian Football League-themed (AFL) mobile app combining psychoeducational modules that teach behavioral strategies derived from acceptance and commitment therapy and positive psychology, social connection, and gamification and casual video games that aims to promote conversations around well-being in young Australian males and fans of AFL.	Well-being	Customization<br>Exploratory or open-world approach<br>Includes minigame<br>Levels or progress feedback<br>Points or scoring<br>Quests or challenges<br>Rewards or prizes<br>Social comparison<br>Social competition<br>Social cooperation<br>Social networking

MindTrails [38]	A proposed redesign of an existing Web-based anxiety training program that includes gamification.	Anxiety disorders	Levels or progress feedback<br>Points or scoring
MoodMission [39]	A mobile app aimed at all age groups that gives its users "missions" (tasks based on cognitive behavioral therapy strategies) to help them deal with mood- and anxiety-related issues.	Anxiety disorders<br>Depressive disorders	Badges or achievements<br>Games of chance<br>Levels or progress feedback<br>Personalization<br>Quests or challenges<br>Rewards or prizes
MOPOrtal/Clans of Oulu [40, 41]	An intervention aiming to encourage physical activity in young Finnish men. In addition to personalized goals, information, and feedback, the intervention contains a location-based mixed-reality game that encourages any kind of activity (physical, social, etc.).	Physical health with mental health and well-being outcomes	Badges or achievements<br>Customization<br>Levels or progress feedback<br>Personalization<br>Rewards or prizes<br>Social networking
Oiva [42]	A mobile app based on acceptance and commitment therapy that teaches skills to manage stress and improve mental wellness.	Well-being	Exploratory or open-world approach<br>Levels or progress feedback<br>Rewards or prizes
REACH App [43]	A mobile app adapting the REACH protocol for childhood anxiety.	Anxiety disorders	Badges or achievements<br>Includes mini-game<br>Levels or progress feedback<br>Narrative or theme<br>Quests or challenges<br>Rewards or prizes<br>Unlockable content
Readysetgoals [44]	A mobile app that supports therapeutic goal setting in the context of treating substance addiction using the metaphor of mountain climbing. Users select activities to do (goals or challenges), wagering points and setting a time limit. Their payoff is determined by the time limit and the difficulty of the task. The more goals users complete, the higher rank they achieve, and the further up the mountain they progress.	Drug use disorders	Artificial challenge<br>Customization<br>Games of chance<br>Levels or progress feedback<br>Narrative or theme<br>Points or scoring<br>Quests or challenges<br>Rewards or prizes

Recovery Record [45]	A mobile app that contains a meal diary, meal reminders, affirmations, rewards, and the functionality to link with clinicians.	Eating disorders	Personalization<br>Rewards or prizes
RehabMaster [46, 47]	A game-based virtual reality rehabilitation program for patients with chronic hemiparetic stroke. It aims to improve health-related quality of life, depression symptoms, and upper extremity function.	Physical health with mental health and well-being outcomes	Customization<br>Includes mini-game<br>Levels or progress feedback<br>Points or scoring<br>Social competition
Shots [15, 48]	A gamified cognitive bias modification of attention task presented as a slot machine. It was emphasized to participants that despite its appearance, it has no gambling elements.	Alcohol use disorders	Levels or progress feedback<br>Narrative or theme<br>Points or scoring<br>Rewards or prizes
SIGMA [49]	An mHealth intervention aimed at overweight young people with maladaptive eating attitudes and behaviors. It contains both an explicit, cognitive-behavioral training component as well as an implicit, attentional bias retraining component.	Eating disorders	Artificial challenge<br>Games of chance<br>Levels or progress feedback<br>Narrative or theme<br>Points or scoring<br>Rewards or prizes<br>Social comparison
Sigrid-Secrets Art Experience [50]	A gamified location-based art experience (geocaching) in Pori, Finland, that aims to promote its users to engage physically and socially with the displayed artworks, contributing to improved well-being.	Well-being	Includes mini-game<br>Narrative or theme<br>Points or scoring<br>Social networking
Sleep Ninja [51]	A mobile app that delivers cognitive behavioral therapy for insomnia to young people. In the app, young people interact with a 'Sleep Ninja', who coaches them through multiple psychoeducational modules to become a 'black belt' in sleep.	Sleep	Customization<br>Exploratory or open-world approach<br>Levels or progress feedback<br>Narrative or theme<br>Personalization
Sleepy Bird [52]	A gamified mobile alarm clock app containing an integrated casual video game. Sleepy Bird rewards participants with 'lives' (i.e. chances to play the casual video game) if they report healthy sleep-wake behaviors.	Sleep<br>Well-being	Artificial challenge<br>Includes mini-game<br>Levels or progress feedback<br>Narrative or theme<br>Personalization

			Points or scoring<br>Rewards or prizes<br>Social competition
SmartCAT [53]	A multi-platform intervention delivering CBT to children experiencing anxiety that consists of a mobile app and a Web-based, integrated clinician portal. SmartCAT contains multiple modules with mini-games and a reward system including both virtual and real-world rewards (agreed on with their clinician).	Anxiety disorders	Badges or achievements<br>Customization<br>Includes mini-game<br>Levels or progress feedback<br>Personalization<br>Points or scoring<br>Rewards or prizes
SOmNI [54]	A mobile app for adolescents that works in conjunction with an activity tracker to increase night-time sleeping hours.	Sleep	Levels or progress feedback<br>Personalization<br>Points or scoring<br>Rewards or prizes<br>Social competition
Stress Free [3]	A mobile app delivering interactive game-based cognitive behavioral therapy (CBT) that targets anxiety and stress in general. A virtual therapist teaches the user generic CBT skills and relaxation techniques.	Anxiety disorders	Includes mini-game<br>Quests or challenges
Superbetter [55, 56]	A multi-platform initiative aiming to gamify resilience and mental health. SuperBetter is made up of a mobile app, a website, and a Web-based forum. Users select well-being-related challenges to complete and are rewarded with points and level-ups when they report completion.	Depressive disorders<br>Well-being	Customization<br>Levels or progress feedback<br>Narrative or theme<br>Points or scoring<br>Quests or challenges<br>Social comparison<br>Social networking
The Wellbeing Game [57]	A free online game that adapts the Five Ways to Wellbeing framework to a game format.	Well-being	Badges or achievements<br>Levels or progress feedback<br>Points or scoring<br>Rewards or prizes<br>Social competition<br>Social cooperation

Therapeutic Evaluative Conditioning [58]	A mobile app that aims to reduce self-aversion and increase aversion to self-injuring behaviors through a short game-like paradigm.	Self-injury or suicide	Artificial challenge<br>Games of chance<br>Points or scoring
This Is Your Life! [59, 60]	A Web-based well-being intervention that uses the metaphor of a professor helping the user journey through various locations towards a flourishing life.	Well-being	Badges or achievements<br>Levels or progress feedback<br>Narrative or theme
Unified Health Gamification [61]	A theory-based, responsive approach enabling application of gamification to multiple facets of health and well-being (physical, social, mental, and cognitive). It aims to allow individual users to participate in a broader social context and interact with people who may have different health interests to their own.	Well-being	Badges or achievements<br>Customization<br>Includes mini-game<br>Levels or progress feedback<br>Personalization<br>Points or scoring<br>Quests or challenges<br>Rewards or prizes<br>Social competition<br>Social cooperation<br>Social networking
Unnamed gamified ABMT intervention [62]	A gamified attentional bias modification paradigm presented as a mobile app. This intervention features cartoon imagery (blue sprites hiding in grass; different colored jewels used as performance feedback).	Anxiety disorders	Levels or progress feedback<br>Narrative or theme<br>Points or scoring
Unnamed gamified nightlife intervention [63]	A gamified typical nightlife intervention delivered in quieter areas of clubbing or music events. It aims to increase awareness of risks associated with substance use.	Alcohol use disorders<br>Drug use disorders	Customization<br>Games of chance<br>Includes mini-game<br>Levels or progress feedback<br>Narrative or theme<br>Points or scoring
Unnamed gamified 'snap' ABMT [64]	An attentional bias modification dot probe task presented as a game of snap where players have to identify whether photographs of two human faces making different expressions depict the same person. Participants' performance is presented as a score and they are encouraged to improve their high score.	Anxiety disorders	Games of chance<br>Levels or progress feedback<br>Narrative or theme<br>Points or scoring

Unnamed mobile app to reduce cognitive vulnerability and mild depressive symptoms [65]	A mobile app that delivers rational emotive behavior therapy aimed at addressing users' cognitive vulnerability and mild depressive symptoms. The app displays a slowly decreasing "Energy" level. Users can increase (i.e. maintain) their "Energy" level by reading informative articles and completing set tasks.	Depressive disorders	Customization<br>Levels or progress feedback<br>Narrative or theme<br>Quests or challenges<br>Rewards or prizes
Unnamed truck driving simulator [66, 67]	A virtual reality action-cue exposure therapy aimed at current or former truck drivers with PTSD from work-related accidents. In addition to the exposure therapy, users can also customize their truck.	Anxiety disorders	Customization<br>Exploratory or open-world approach<br>Games of chance<br>Levels or progress feedback<br>Narrative or theme<br>Rewards or prizes
VIMSE/Itsy [68, 69]	A self-directed, gamified, virtual reality exposure therapy application aimed at spider phobia.	Anxiety disorders	Customization<br>Levels or progress feedback<br>Narrative or theme
Woebot [70]	A mobile app containing a text-based conversational agent that delivers CBT microsessions.	Anxiety disorders<br>Depressive disorders	Includes mini-game<br>Personalization

References

1. Donker T, Van Esveld S, Fischer N, Van Straten A. 0Phobia-towards a virtual cure for acrophobia: study protocol for a randomized controlled trial. Trials. 2018 Aug;19. PMID: WOS:000441361500002. doi: 10.1186/s13063-018-2704-6.
2. Edney S, Plotnikoff R, Vandelanotte C, Olds T, De Bourdeaudhuij I, Ryan J, et al. "Active Team" a social and gamified app-based physical activity intervention: randomised controlled trial study protocol. BMC Public Health. 2017 Nov 2;17(1):859. PMID: 29096614. doi: 10.1186/s12889-017-4882-7.
3. Christoforou M, Sáez Fonseca AJ, Tsakanikos E. Two Novel Cognitive Behavioral Therapy-Based Mobile Apps for Agoraphobia: Randomized Controlled Trial. J Med Internet Res. 2017 11/24;19(11):e398. doi: 10.2196/jmir.7747.
4. Harris MA. The relationship between physical inactivity and mental wellbeing: Findings from a gamification-based community-wide physical activity intervention. Health Psychol Open. 2018 Jan-Jun;5(1):2055102917753853. PMID: 29372067. doi: 10.1177/2055102917753853.
5. Hunter RF, de Silva D, Reynolds V, Bird W, Fox KR. International inter-school competition to encourage children to walk to school: a mixed methods feasibility study. BMC research notes. 2015 Jan 27;8:19. PMID: 25623073. doi: 10.1186/s13104-014-0959-x.
6. Coombes E, Jones A. Gamification of active travel to school: A pilot evaluation of the Beat the Street physical activity intervention. Health Place. 2016 May;39:62-9. PMID: 26974232. doi: 10.1016/j.healthplace.2016.03.001.
7. Intelligent Health. Building Active Communities: Beat the Street. 2016 [cited 2018 December 6]; Available from: http://www.intelligenthealth.co.uk/wp-content/uploads/2015/09/IH-Corporate-Brochure-3.pdf. Archived at http://www.webcitation.org/74SHEXZcN.
8. Fox LG, Barnes D. BoosterBuddy: Using Gamification as a Compensatory Strategy for Motivational Deficits. Psychiatric Services. 2016;67(1):141-2. doi: http://dx.doi.org/10.1176/appi.ps.661101.
9. Milward J, Deluca P, Drummond C, Watson R, Dunne J, Kimergård A. Usability Testing of the BRANCH Smartphone App Designed to Reduce Harmful Drinking in Young Adults. JMIR mHealth and uHealth. 2017 2017/08/08;5(8):e109. doi: 10.2196/mhealth.7836.
10. Boyle SC, Earle AM, LaBrie JW, Smith DJ. PNF 2.0? Initial evidence that gamification can increase the efficacy of brief, web-based personalized normative feedback alcohol interventions. Addictive Behaviors. 2017 2017/04/01/;67:8-17. doi: https://doi.org/10.1016/j.addbeh.2016.11.024.
11. Earle AM, LaBrie JW, Boyle SC, Smith D. In pursuit of a self-sustaining college alcohol intervention: Deploying gamified PNF in the real world. Addictive Behaviors. 2018 2018/05/01/;80:71-81. doi: https://doi.org/10.1016/j.addbeh.2018.01.005.
12. Miloff A, Marklund A, Carlbring P. The challenger app for social anxiety disorder: New advances in mobile psychological treatment. Internet Interventions. 2015 2015/11/01/;2(4):382-91. doi: https://doi.org/10.1016/j.invent.2015.08.001.
13. Boettcher J, Magnusson K, Marklund A, Berglund E, Blomdahl R, Braun U, et al. Adding a smartphone app to internet-based self-help for social anxiety: A randomized controlled trial. Computers in Human Behavior. 2018 2018/10/01/;87:98-108. doi: https://doi.org/10.1016/j.chb.2018.04.052.
14. Boendermaker WJ, Boffo M, Wiers RW. Exploring Elements of Fun to Motivate Youth to Do Cognitive Bias Modification. Games for Health Journal. 2015 2015/12/01;4(6):434-43. doi: 10.1089/g4h.2015.0053.
15. Boendermaker WJ, Prins PJM, Wiers RW. Cognitive Bias Modification for adolescents with substance use problems – Can serious games help? Journal of Behavior Therapy and Experimental Psychiatry. 2015 2015/12/01/;49:13-20. doi: https://doi.org/10.1016/j.jbtep.2015.03.008.

16. Gottlieb J, Harper Romero K, Penn D, Mueser K, Chiko B. Web-based cognitive-behavioral therapy for auditory hallucinations in persons with psychosis: "Coping with voices". Schizophrenia Bulletin. 2013;39(SUPPL. 1):S288-S9. doi: http://dx.doi.org/10.1093/schbul/sbt011.
17. Gottlieb JD, Gidugu V, Maru M, Tepper MC, Davis MJ, Greenwold J, et al. Randomized controlled trial of an internet cognitive behavioral skills-based program for auditory hallucinations in persons with psychosis. Psychiatric rehabilitation journal. 2017 Sep;40(3):283-92. PMID: 28517948. doi: 10.1037/prj0000258.
18. Struik LL, Bottorff JL, Baskerville NB, Oliffe JL. The Crush the Crave Quit Smoking App and Young Adult Smokers: Qualitative Case Study of Affordances. JMIR mHealth and uHealth. 2018 2018/06/08;6(6):e134. doi: 10.2196/mhealth.9489.
19. Baskerville NB, Struik LL, Guindon GE, Norman CD, Whittaker R, Burns C, et al. Effect of a Mobile Phone Intervention on Quitting Smoking in a Young Adult Population of Smokers: Randomized Controlled Trial. JMIR mHealth and uHealth. 2018 2018/10/23;6(10):e10893. doi: 10.2196/10893.
20. Baskerville NB, Struik LL, Hammond D, Guindon GE, Norman CD, Whittaker R, et al. Effect of a mobile phone intervention on quitting smoking in a young adult population of smokers: randomized controlled trial study protocol. JMIR research protocols. 2015 Jan 19;4(1):e10. PMID: 25599695. doi: 10.2196/resprot.3823.
21. Cobb NK, Poirier J. Effectiveness of a Multimodal Online Well-Being Intervention: A Randomized Controlled Trial. American Journal of Preventive Medicine. 2014 2014/01/01/;46(1):41-8. doi: https://doi.org/10.1016/j.amepre.2013.08.018.
22. Ben-Moussa M, Rubo M, Debracque C, Lange W-G. DJINNI: A Novel Technology Supported Exposure Therapy Paradigm for SAD Combining Virtual Reality and Augmented Reality. Frontiers in psychiatry. 2017;8:26-. PMID: 28503155. doi: 10.3389/fpsyt.2017.00026.
23. Choo A, May A, editors. Virtual mindfulness meditation: Virtual reality and electroencephalography for health gamification. 2014 IEEE Games Media Entertainment; 2014 22-24 Oct. 2014.
24. Vahabzadeh A, Keshav NU, Salisbury JP, Sahin NT. Improvement of Attention-Deficit/Hyperactivity Disorder Symptoms in School-Aged Children, Adolescents, and Young Adults With Autism via a Digital Smartglasses-Based Socioemotional Coaching Aid: Short-Term, Uncontrolled Pilot Study. JMIR mental health. 2018 2018/04/02;5(2):e25. doi: 10.2196/mental.9631.
25. Pieters EK, De Raedt R, Enock PM, De Putter LMS, Braham H, McNally RJ, et al. Examining a Novel Gamified Approach to Attentional Retraining: Effects of Single and Multiple Session Training. Cognitive Therapy and Research. 2017 2017/02/01;41(1):89-105. doi: 10.1007/s10608-016-9803-z.
26. Mohr DC, Tomasino KN, Lattie EG, Palac HL, Kwasny MJ, Weingardt K, et al. IntelliCare: An Eclectic, Skills-Based App Suite for the Treatment of Depression and Anxiety. J Med Internet Res. 2017 2017/01/05;19(1):e10. doi: 10.2196/jmir.6645.
27. Ciocarlan A, Masthoff J, editors. Kindness is contagious: Exploring engagement in a gamified persuasive intervention for wellbeing. 2017: CEUR-WS.
28. Ciocarlan A, Masthoff J, Oren N, editors. Kindness is contagious: Study into exploring engagement and adapting persuasive games for wellbeing. 2018: Association for Computing Machinery, Inc.
29. Ciocarlan A, Masthoff J, Oren N, editors. Kindness is contagious: Demonstration of a persuasive intervention for wellbeing. 2018: Association for Computing Machinery, Inc.
30. Vilardaga R, Rizo J, Zeng E, Kientz JA, Ries R, Otis C, et al. User-Centered Design of Learn to Quit, a Smoking Cessation Smartphone App for People With Serious Mental Illness. JMIR serious games. 2018 Jan 16;6(1):e2. PMID: 29339346. doi: 10.2196/games.8881.

31. Uzuegbunam N, Wing-Hang W, Cheung SS, Ruble L, editors. MEBook: Kinect-based self-modeling intervention for children with autism. 2015 IEEE International Conference on Multimedia and Expo (ICME); 2015 29 June-3 July 2015.
32. Uzuegbunam N, Wong WH, Cheung SS, Ruble L. MEBook: Multimedia Social Greetings Intervention for Children with Autism Spectrum Disorders. IEEE Transactions on Learning Technologies. 2018:1-. doi: 10.1109/TLT.2017.2772255.
33. Cheng VWS, editor. Studying the effectiveness of game-based solutions in a wellbeing app. 2017: Association for Computing Machinery, Inc.
34. Cheng VWS, Davenport TA, Johnson D, Vella K, Mitchell J, Hickie IB. An App That Incorporates Gamification, Mini-Games, and Social Connection to Improve Men's Mental Health and Well-Being (MindMax): Participatory Design Process. JMIR mental health. 2018 2018/11/19;5(4):e11068. doi: 10.2196/11068.
35. Vella K, Cheng VWS, Johnson D, Davenport T, Peever N, Mitchell J, editors. Motivating engagement with a wellbeing app using video games and gamification. 2017: CEUR-WS.
36. Vella K, Peever N, Klarkowski M, Ploderer B, Mitchell J, Johnson D. Using Applied Games to Engage mHealth Users: A Case Study of MindMax. Proceedings of the 2018 Annual Symposium on Computer-Human Interaction in Play; Melbourne, VIC, Australia: ACM; 2018.
37. Mitchell J, Vella K, Johnson D, Peever N, Cheng VWS, Davenport T, et al. MindMax: Using Videogames and Sport to Engage Young Men and Improve Wellbeing. 2nd Symposium Computing and Mental Health; Denver, CO2017.
38. Stevens R, Polk K, Merrill C, Feng F, Weiss M, Brosnan E, et al., editors. User experience design to enhance the effectiveness of mobile technologies for the treatment of mental health. 2018: Institute of Electrical and Electronics Engineers Inc.
39. Bakker D, Kazantzis N, Rickwood D, Rickard N. Development and Pilot Evaluation of Smartphone-Delivered Cognitive Behavior Therapy Strategies for Mood- and Anxiety-Related Problems: MoodMission. Cognitive and Behavioral Practice. 2018 2018/11/01/;25(4):496-514. doi: https://doi.org/10.1016/j.cbpra.2018.07.002.
40. Ahola R, Pyky R, Jämsä T, Mäntysaari M, Koskimäki H, Ikäheimo TM, et al. Gamified physical activation of young men--a Multidisciplinary Population-Based Randomized Controlled Trial (MOPO study). BMC public health. 2013;13:32. PMID: CN-00853604. doi: 10.1186/1471-2458-13-32.
41. Pyky R, Koivumaa-Honkanen H, Leinonen A-M, Ahola R, Hirvonen N, Enwald H, et al. Effect of tailored, gamified, mobile physical activity intervention on life satisfaction and self-rated health in young adolescent men: A population-based, randomized controlled trial (MOPO study). Computers in Human Behavior. 2017 2017/07/01/;72:13-22. doi: https://doi.org/10.1016/j.chb.2017.02.032.
42. Ahtinen A, Mattila E, Välkkynen P, Kaipainen K, Vanhala T, Ermes M, et al. Mobile Mental Wellness Training for Stress Management: Feasibility and Design Implications Based on a One-Month Field Study. JMIR mHealth and uHealth. 2013 2013/07/10;1(2):e11. doi: 10.2196/mhealth.2596.
43. Patwardhan M, Stoll R, Hamel DB, Amresh A, Gary KA, Pina A. Designing a mobile application to support the indicated prevention and early intervention of childhood anxiety. Proceedings of the conference on Wireless Health; Bethesda, Maryland. 2811954: ACM; 2015. p. 1-8.
44. Siriaraya P, Visch V, Dooren MMMv, Spijkerman R, editors. Learnings and Challenges in Designing Gamifications for Mental Healthcare: The Case Study of the Readysetgoals Application. 2018 10th International Conference on Virtual Worlds and Games for Serious Applications (VS-Games); 2018 5-7 Sept. 2018.
45. Lindgreen P, Lomborg K, Clausen L. Patient Experiences Using a Self-Monitoring App in Eating Disorder Treatment: Qualitative Study. JMIR mHealth and uHealth. 2018 2018/06/22;6(6):e10253. doi: 10.2196/10253.

46. Shin JH, Bog Park S, Ho Jang S. Effects of game-based virtual reality on health-related quality of life in chronic stroke patients: A randomized, controlled study. Computers in Biology and Medicine. 2015;63:92-8. doi: 10.1016/j.compbiomed.2015.03.011.
47. Shin J-H, Ryu H, Jang SH. A task-specific interactive game-based virtual reality rehabilitation system for patients with stroke: a usability test and two clinical experiments. Journal of NeuroEngineering and Rehabilitation. 2014 2014/03/06;11(1):32. doi: 10.1186/1743-0003-11-32.
48. Boendermaker WJ, Sanchez Maceiras S, Boffo M, Wiers RW. Attentional Bias Modification With Serious Game Elements: Evaluating the Shots Game. JMIR serious games. 2016 2016/12/06;4(2):e20. doi: 10.2196/games.6464.
49. Podina IR, Fodor LA, Cosmoiu A, Boian R. An evidence-based gamified mHealth intervention for overweight young adults with maladaptive eating habits: study protocol for a randomized controlled trial. Trials. 2017 Dec 12;18(1):592. PMID: 29233169. doi: 10.1186/s13063-017-2340-6.
50. Ihamäki P, Heljakka K, editors. The sigrid-secrets geocaching trail: Influencing well-being through a gamified art experience. 2017: CEUR-WS.
51. Werner-Seidler A, O'Dea B, Shand F, Johnston L, Frayne A, Fogarty AS, et al. A Smartphone App for Adolescents With Sleep Disturbance: Development of the Sleep Ninja. JMIR mental health. 2017 2017/07/28;4(3):e28. doi: 10.2196/mental.7614.
52. Ilhan E, Sener B, Hacihabiboğlu H. Creating awareness of sleep-wake hours by gamification. In: Tscheligi M, De Ruyter B, Meschtscherjakov A, Fuchsberger V, Murer M, Tscheligi M, editors.: Springer Verlag; 2016. p. 122-33.
53. Pramana G, Parmanto B, Lomas J, Lindhiem O, Kendall PC, Silk J. Using Mobile Health Gamification to Facilitate Cognitive Behavioral Therapy Skills Practice in Child Anxiety Treatment: Open Clinical Trial. JMIR serious games. 2018 2018/05/10;6(2):e9. doi: 10.2196/games.8902.
54. Stremler R, Vaidyanathan P, Cafazzo J, Goyal S, Katzman DK, Keilty K, et al. Design, development and feasibility testing of an mhealth application for sleep-restricted adolescents. Sleep Medicine. 2017;40(Supplement 1):e315-e6.
55. Chou T, Bry LJ, Comer JS. Multimedia Field Test: Evaluating the Creative Ambitions of SuperBetter and Its Quest to Gamify Mental Health. Cognitive and Behavioral Practice. 2017 2017/02/01/;24(1):115-20. doi: https://doi.org/10.1016/j.cbpra.2016.10.002.
56. Roepke AM, Jaffee SR, Riffle OM, McGonigal J, Broome R, Maxwell B. Randomized Controlled Trial of SuperBetter, a Smartphone-Based/Internet-Based Self-Help Tool to Reduce Depressive Symptoms. Games Health J. 2015 Jun;4(3):235-46. PMID: 26182069. doi: 10.1089/g4h.2014.0046.
57. Keeman A, Näswall K, Malinen S, Kuntz J. Employee Wellbeing: Evaluating a Wellbeing Intervention in Two Settings. Frontiers in Psychology. 2017 2017-April-04;8(505). doi: 10.3389/fpsyg.2017.00505.
58. Franklin JC, Fox KR, Franklin CR, Kleiman EM, Ribeiro JD, Jaroszewski AC, et al. A brief mobile app reduces nonsuicidal and suicidal self-injury: Evidence from three randomized controlled trials. Journal of Consulting and Clinical Psychology. 2016;84(6):544-57. doi: 10.1037/ccp0000093.
59. Kelders SM, Sommers-Spijkerman M, Goldberg J. Investigating the Direct Impact of a Gamified Versus Nongamified Well-Being Intervention: An Exploratory Experiment. Journal of Medical Internet Research. 2018 Jul;20(7). PMID: WOS:000440103600001. doi: 10.2196/jmir.9923.
60. Ludden GDS, Kelders SM, Snippert BHJ, editors. 'This Is Your Life!'. Persuasive Technology; 2014 2014//; Cham: Springer International Publishing.
61. Shahrestani A, Gorp PV, Blanc PL, Greidanus F, Groot Kd, Leermakers J, editors. Unified Health Gamification can significantly improve well-being in corporate environments. 2017 39th Annual International Conference of the IEEE Engineering in Medicine and Biology Society (EMBC); 2017 11-15 July 2017.
62. Dennis TA, O'Toole LJ. Mental health on the go: Effects of a gamified attention-bias modification mobile application in trait-anxious adults. Clinical Psychological Science. 2014 2014/09/01;2(5):576-90. doi: 10.1177/2167702614522228.

63. Gamberini L, Spagnolli A, Nucci M, DeGiuli G, Villa C, Monarca V, et al. A gamified solution to brief interventions for nightlife well-being. In: Tscheligi M, De Ruyter B, Meschtscherjakov A, Fuchsberger V, Murer M, Tscheligi M, editors.: Springer Verlag; 2016. p. 230-41.
64. Notebaert L, Clarke PJF, Grafton B, MacLeod C. Validation of a novel attentional bias modification task: The future may be in the cards. Behaviour Research and Therapy. 2015 2015/02/01/;65:93-100. doi: https://doi.org/10.1016/j.brat.2014.12.007.
65. Giosan C, Mogoase C, Cobeanu O, Szentagotai Tatar A, Muresan V, Boian R. Using a smartphone app to reduce cognitive vulnerability and mild depressive symptoms: Study protocol of an exploratory randomized controlled trial. Trials. 2016 Dec 28;17(1):609. PMID: 28031038. doi: 10.1186/s13063-016-1740-3.
66. Ecrepont A, Haidon C, Girard B, Menelas BJ, editors. A fully customizable truck-driving simulator for the care of people suffering from Post-Traumatic Stress Disorder. 2016 IEEE International Conference on Serious Games and Applications for Health (SeGAH); 2016 11-13 May 2016.
67. Menelas B-AJ, Haidon C, Ecrepont A, Girard B. Use of virtual reality technologies as an Action-Cue Exposure Therapy for truck drivers suffering from Post-Traumatic Stress Disorder. Entertainment Computing. 2018 2018/01/01/;24:1-9. doi: https://doi.org/10.1016/j.entcom.2017.10.001.
68. Lindner P, Miloff A, Hamilton W, Reuterskiöld L, Andersson G, Powers MB, et al. Creating state of the art, next-generation Virtual Reality exposure therapies for anxiety disorders using consumer hardware platforms: design considerations and future directions. Cognitive Behaviour Therapy. 2017 2017/09/03;46(5):404-20. doi: 10.1080/16506073.2017.1280843.
69. Miloff A, Lindner P, Hamilton W, Reuterskiöld L, Andersson G, Carlbring P. Single-session gamified virtual reality exposure therapy for spider phobia vs. traditional exposure therapy: study protocol for a randomized controlled non-inferiority trial. Trials. 2016 2016/02/02;17(1):60. doi: 10.1186/s13063-016-1171-1.
70. Fitzpatrick KK, Darcy A, Vierhile M. Delivering Cognitive Behavior Therapy to Young Adults With Symptoms of Depression and Anxiety Using a Fully Automated Conversational Agent (Woebot): A Randomized Controlled Trial. JMIR Ment Health. 2017 06/06;4(2):e19. doi: 10.2196/mental.7785.
